# Functional response of *Franklinothrips vespiformis* (Thysanoptera: Aeolothripidae) to eggs and nymphs of *Bemisia tabaci* (Hemiptera: Aleyrodidae)

**DOI:** 10.1093/jisesa/ieae030

**Published:** 2024-03-05

**Authors:** Erich N Schoeller, Joshua Hogan, Cindy L McKenzie, Lance S Osborne

**Affiliations:** Department of Entomology, University of Georgia, Griffin, GA 30223, USA; Department of Entomology and Nematology, Mid-Florida Research and Education Center, University of Florida, Apopka, Florida, USA; United States Horticultural Research Laboratory, United States Department of Agriculture, Agricultural Research Service, Fort Pierce, FL, 34945USA; Department of Entomology and Nematology, Mid-Florida Research and Education Center, University of Florida, Apopka, Florida, USA

**Keywords:** natural enemy, biological control, integrated pest management, trophic ecology, whitefly

## Abstract

The Middle East Asia Minor 1 biotype of *Bemisia tabaci* Gennadius (Hemiptera: Aleyrodidae) is a greenhouse and field crop pest of global significance. The objective of this study was to assess the potential of the generalist predatory thrips, *Franklinothrips vespiformis* Crawford (Thysanoptera: Aeolothripidae), as a biological control agent for *B. tabaci*. This was achieved by determining the functional responses of *F. vespiformis* larvae and adults to the egg and nymphal stages of *B. tabaci* under laboratory conditions. Analyses consisted of 10 replicates of each predator and prey stage combination on bean leaf discs for a 24-h period. Following logistic regression analyses to determine the functional response type exhibited, response parameters were estimated with nonlinear least squares regression using Roger’s equation. Results showed that *F. vespiformis* larvae and adults exhibited a Type II functional response when feeding on immature *B. tabaci*. The handling times (*T*_*h*_) of *F. vespiformis* larvae and adults were magnitudes higher for *B. tabaci* nymphs than they were for eggs, which were in part driven by the higher attack rates (*a*) observed on eggs. The maximum attack rate (*T*/*T*_*h*_) for *B. tabaci* eggs and nymphs exhibited by first-stage larvae, second-stage larvae, and adult *F. vespiformis* increased with increasing predator age. Results from this study suggest that *F. vespiformis* larvae and particularly adults are promising biological control agents for *B. tabaci* and are efficient predators at both low and high prey densities.

## Introduction

The whitely, *Bemisia tabaci* (Gennadius) (Hemiptera: Aleyrodidae), is a highly polyphagous pest recorded on over 1,000 plant species in 74 families, of which many are economically important field and greenhouse crops (Oliveira et al. 2001, Simmons et al. 2008, Li et al. 2021). Adding to the management complexities arising from its broad host range is the fact that *B. tabaci* is a cryptic species complex comprised of over 44 genetically distinct yet morphologically indistinguishable species (Brown et al. 1995, De Barro et al. 2011, Malka et al. 2018, Kanakala and Ghanim 2019). The B biotype, also known as Middle East Asia Minor 1 (MEAM1), is one of the most economically important species in this complex. Widely spread by trade throughout North and South America in the late 1980s, MEAM1 rapidly became a major agricultural pest in these regions (Broadbent et al. 1989, Elfekih et al. 2018, Li et al. 2021). Now, after nearly 40 yr since its spread across the Americas, the feeding activity of MEAM1 causes significant economic losses to global crop production, reaching billions of USD annually (Henneberry and Faust 2008, Stansly and Naranjo 2010, Pozebon et al. 2020). Crop losses incurred by *B. tabaci* feeding are often attributed to both lower yields, a result of reduced nutrient availability, and the negative effects caused by a large number of plant pathogens that *B. tabaci* vectors (Jones 2003, Polston et al. 2014, Gilbertson et al. 2015).

In recent years, *B. tabaci* has developed resistance to several major insecticide classes, such as organophosphates, pyrethroids, and neonicotinoids (Naveen et al. 2017, Wang et al. 2023). This has resulted in a marked decrease in the effectiveness of traditional chemical-based control strategies (Castle and Prabhaker 2013, Dângelo et al. 2018, Horowitz et al. 2020). The rise of insecticide-resistant adaptations in agricultural pests such as *B. tabaci* represents an existential threat to growers who lack alternatives to chemical pesticides, especially in countries with agriculture-dependent economies (Hu 2020, Van den Berg et al. 2022). These growers are forced to overuse ineffective chemicals to meet food production goals and often do so with minimal training on how to safely mix, apply, or dispose of these chemicals (Wang et al. 2018, Matowo et al. 2020). The consequences of these practices are well documented, and great harm is often exacted on growers, local ecosystems, and nearby communities (Desnuex et al. 2007, Damalas and Eleftherohorinos 2011, Kim et al. 2017, Major et al. 2018, Gunstone et al. 2021). Implementation of integrated pest management (IPM) practices became a focus in the 1960s in response to the overdependence on pesticides and the threat they represent (Stern et al. 1959, Kogan 1998). Work invested into the framework of IPM since its inception has resulted in novel alternative pest control strategies that are constantly refined, proven, and utilized by growers worldwide (Singh et al. 2020, Kvakkestad et al. 2021).

Biological control is an important component of IPM that relies on the introduction of natural enemies to manage pest populations below economically damaging thresholds. Biological control has a proven history of success in *B. tabaci* management programs (Gerling 1986, Goolsby et al. 2004, Liu et al. 2015, Sani et al. 2020). Multiple biological control agents are available commercially for *B. tabaci* control in the United States. Some of these agents include phytoseiid mites (*Amblydromalus limonicus* Garman and McGregor, *Amblyseius swirskii* Athias-Henriot, *Neoseiulus cucumeris* Oudemans), parasitoids (*Encarsia formosa* Gahan, *Eretmocerus eremicus* Rose and Zolnerowich), predatory bugs (*Dicyphus hesperus* Knight, *Nesidiocoris tenuis* (Reuter), *Orius insidiosus* [Say]), lacewings (*Chrysoperla* spp., *Sympherobius barberi* Banks), ladybird beetles (*Delphastus catalinae* [Horn], *Delphastus pusillus* [LeConte]), entomopathogenic fungi (*Beauveria bassiana* [Bals.] Vuill., *Isaria fumosoroseus* Wize), and entomopathogenic nematodes (*Heterorhabditis bacteriophora* Poinar, *Steinernema carpocapsae* [Weiser], *Steinernema feltiae* [Filipjev]). The author’s observations over the last few decades have been that many of these commercially available natural enemies will feed on whiteflies but have proven to not be very effective under greenhouse conditions. For example, *E. eremicus* is a prolific host-feeder (Headrick et al. 1995) but has not established well in commercial or research greenhouses in Florida and requires frequent mass releases to achieve desirable results. *Delphastus* spp. only feed on whiteflies and need high populations to maintain active pest control in the greenhouse. Additionally, a strict whitefly diet for green lacewings has been shown to be insufficient for their development (Legaspi et al. 1994). These are just a few examples of the challenges of using some of the currently available commercial biological control agents. Given the broad distribution and host range of *B. tabaci*, there is a need for additional biological control agents that work well under different environmental conditions, production practices, or on crops where certain control agents’ performance may be lacking.

*Franklinothrips vespiformis* Crawford (Thysanoptera: Aeolothripidae) is a species of tropical ant-mimicking thrips known to be a generalist and obligate predator of a wide range of small arthropods worldwide (Loomans and Vierbergen 1999, Mound and Reynaud 2005, Hussain et al. 2022). Though the number of studies that examine the biological control potential of *F. vespiformis* is limited, it has already been successfully tested, marketed, and bred for sale and use against pest thrips in Europe (Cox et al. 2006, Larentzaki et al. 2007b, Pizzol et al. 2008, 2012). No studies exist to our knowledge that have examined the effectiveness of *F. vespiformis* as a biological control agent of *B. tabaci*, but it has been observed to predate nymphal stages of *B. tabaci* in the laboratory and was recorded as a natural enemy of whiteflies in South America (Moulton 1932, Arakaki and Okajima 1998). Observations of *F. vespiformis* being a potential whitefly predator, along with its demonstrated effectiveness on other pest species in Europe, have generated interest in determining the suitability of *F. vespiformis* as a biological control agent of *B. tabaci*.

The rate at which a predator kills its prey at different prey densities, also known as its functional response, is an important component of predator–prey population dynamics (Murdoch and Oaten 1975). While considered by some as insufficient as a sole determinant for selecting biological control agents (Waage 1990, Cuthbert et al. 2018), it remains a useful metric in assessing the potential of natural enemies in regulating prey populations (Houck and Strauss 1985, Fernández-Arhex and Corley 2003). Holling (1959) proposed 3 types of functional responses: Type I response describes a linear relationship between prey consumption and increasing prey density; Type II response describes a hyperbolic negative density-dependent relationship where predation rate decreases with increasing prey density due to time limitations, not prey availability; and Type III response describes a sigmoidal-positive density dependence where predation rate first increases at lower prey density and then decreases at higher prey density. Functional response type and parameters are influenced by many factors, such as prey stage and size (Kalinoski and DeLong 2016, Uiterwaal et al. 2017), so to evaluate the potential of *F. vespiformis* as a biological control agent of *B. tabaci*, we assessed the functional responses of different *F. vespiformis* life stages to *B. tabaci* eggs and nymphs of varying densities under laboratory conditions.

## Materials and Methods

### Host Plants

Lima beans (*Phaseolus lunatus* L. cv. ‘Henderson Bush’) were used as host plants to rear *B. tabaci* (MEAM1) colonies and provide leaf disks for experimental trials. Plants were initiated in 6-cm-diameter plastic pots containing Professional Growing Mix (Sun Gro Horticulture, Bellevue, Washington). Plants were fertilized with 5 g (medium label rate) of Osmocote Plus (15-9-12 N-P-K) (Scotts-Sierra Horticultural Products Co., Marysville, Ohio) per pot. Pots were held within insect-free cages (60 cm dimensions, 300 µm aperture) that were isolated by elevating them on pots over water under greenhouse conditions (28 ± 10 °C, 85 ± 10% RH, ambient light conditions) and watered 3 times per week. New growth was pruned to maintain plants at the 2-leaf stage for 2–3 wk until needed for *B. tabaci* colony maintenance or to provide clean leaf discs. Cotton plants (*Gossypium hirsutum* L. cv. ‘Deltapine 1321 B2RF’) were used to maintain greenhouse colonies of *F. vespiformis*. Cotton plants were grown using the same methods described above for lima beans, except cotton plants were not water isolated or pruned at any time during their use.

### Insect Colonies

*Franklinothrips vespiformis.* A colony of *F. vespiformis* originated from individuals collected from low-lying vegetation located on the premises of the University of Florida’s Mid-Florida Research and Education Center (MREC) in Apopka, Florida. The *F. vespiformis* colony was maintained using an open bench-rearing system within a greenhouse (28 ± 10 °C, 85 ± 10% RH) using mature cotton as ovipositional hosts. The *F. vespiformis* colony was fed a diet consisting of decapsulated cysts of the brine shrimp *A. franciscana* (BioBee USA, Atlanta, Georgia) distributed onto the leaves of each cotton plant and supplemented with additional prey of spider mites, *Tetranychus urticae* Koch (Acari: Tetranychidae); chilli thrips, *Scirtothrips dorsalis* Hood (Thysanoptera: Thripidae); and black thrips, *Echinothrips americanus* Morgan (Thysanoptera: Thripidae). Clean cotton plants and supplemental prey plants were rotated in as needed.

To obtain various *F. vespiformis* life stages for functional response trials, adult females of unknown age were aspirated from the greenhouse colony and placed onto square 3 cm^2^ bean leaf pieces suspended in 20 ml of a 2% BiTek agar solution (Difco Labs, Detroit, Michigan) within a 9-cm-diameter Petri dish. A small amount (10 mg) of decapsulated cysts of brine shrimp *Artemia franciscana* Kellogg (Anostraca: Artemiidae) (Biobee USA, Atlanta, Georgia) were added to the surface of leaves as a source of food (Schoeller et al. 2022). Lids with nylon mesh for ventilation were placed onto the dishes, and then dishes were sealed with Parafilm M (Bemis Company Inc., Neenah, Wisconsin) and placed into an environmental chamber and maintained at 25 ± 1 °C and 75 ± 10% RH for 48 h to allow oviposition to occur. Adults were removed and excess cysts were washed off the discs before replacing the lids and placed back in the environmental chamber. Leaves were checked daily for larval emergence (approximately 10 days postoviposition). Newly emerged larvae were transferred to 30-ml solo cups with fine nylon mesh ventilated lids in groups of 10. Larvae were fed a mixed diet of decapsulated *A. franciscana* cysts and *B. tabaci* eggs on 22-mm-diameter lima bean leaf discs. Larvae designated for larvae 1 assays were used within 12 h of emergence. Larvae were transferred to new cups every other day and monitored daily until they were at the desired stage for experiments.

*Bemisia tabaci.* Colonies of *B. tabaci* (MEAM1) used in this study were established and maintained on ca. 2-wk-old lima bean in climate-controlled insectary rooms at MREC and were screened quarterly for biotype confirmation. Beans were used for rearing whiteflies and bioassays as the trichome density on cotton leaves made it difficult to count and manipulate whitefly numbers. The rapid growth of bean plants also facilitated the timing of stages between predator and prey. Plants were replaced with new ones every 6 wk. All climate-controlled rooms were set at 25 ± 2 °C, 75 ± 10% RH and 12:12 h (L:D) photoperiod. Adult *B. tabaci* were aspirated from colonies and introduced into rearing cages (BugDorm2120; MegaView Science Education Services, Taichung, Taiwan) containing one lima bean plant. Cages were kept isolated in the greenhouse for 24–48 h to allow for oviposition before adults were removed. Infested plants were held in isolation until needed. Eggs and nymphs (second and third instar) on these plants were used for functional response experiments after 1 or 10 days postoviposition, respectively. Although adult *B. tabaci* is preyed upon by adult *F. vespiformis* (~5 adults/day when confined within diet cups, E.N. Schoeller, personal observation), the functional response to this stage was not examined, as experimental conditions would not provide reliable data for field or greenhouse conditions where adults can fly to escape predation attempts.

### Functional Response Bioassays

Experimental arenas consisted of 30-ml solo cups with fine nylon mesh ventilated lids. A 20-mm-diameter bean leaf disc taken from experimental colonies was placed at the bottom of each cup with the abaxial side facing up onto 3 ml of 2% agar to maintain freshness. The number of whitefly eggs or nymphs on discs was counted under a dissecting microscope, and individuals were removed until the desired stage presence and densities were reached. Predator treatments consisted of first- and second-stage larvae as well as adults. First-instar *F. vespiformis* were either used within 12 h of emergence or separated into groups of 5 individuals and placed in arenas described above with food and allowed to develop for use in other treatments. Larvae were fed on a mixed diet of *B. tabaci* eggs and decapsulated *A. franciscana* cysts and maintained in environmental chambers (25 ± 2 °C, 75 ± 10% RH, 12:12 h [L:D] photoperiod) until they reached the desired developmental stage.

Based on preliminary trials, different densities of *B. tabaci* were used depending on maximum predation observed for each predator stage and high prey density treatments were included to facilitate locating the asymptote of the response curves. For adult *F. vespiformis* trials, whitefly densities of 25, 50, 75, 100, 125, or 150 eggs and 25, 10, 25, 50, 75, or 100 nymphal stages were used. First- and second-stage *F. vespiformis* larvae trials utilized whitefly densities of 5, 10, 20, 30, 40, or 50 individuals for both the egg and nymph stages. Adult and first-instar *F. vespiformis* were used in experiments within 12 h of emergence from the eggs or pupal cocoons, respectively. Second instar *F. vespiformis* were removed from colonies 15 days after hatching and starved for 24 h before being used in trials. It was not necessary to starve adults as they spent 2–3 days in the pupal cocoons before emerging and were already in a starved state. After 24 h of exposure, predators were removed from arenas, and the number of whitefly eggs or nymphs consumed was recorded. Trials were performed at 25 ± 2 °C, 75 ± 10% RH, and a photoperiod of 12:12 h (L: D) in environmentally controlled chambers. Ten replicates were conducted at each prey density.

### Statistical Analysis

The analysis of *F. vespiformis* functional response on *B. tabaci* involved 2 distinctive steps. The first step discriminated between Type II and Type III functional responses, which was achieved by performing logistic regression of the proportion of prey eaten $\left(N_{e}/N_{0}\right)$ vs. initial prey density $N_{0}$, where *N*_*e*_ is the number of prey consumed, and *N*_0_ is the initial prey density (Trexler et al. 1988, Casas and Hulliger 1994). A polynomial function was fitted to describe the relationship between $\left(N_{e}/N_{0}\right)$ and *N*_0_ where *P*_0_, *P*_1_, *P*_2_, and *P*_3_ are the intercept, linear, quadratic, and cubic coefficients, respectively (Equation 1).


$$\begin{array}{l} \frac{N_{e}}{N_{0}}=\;\frac{exp(P_{0}+P_{1}N_{0}+P_{2}N_{0}^{2}+P_{3}N_{0}^{3})}{1+exp(P_{0}+P_{1}N_{0}+P_{2}N_{0}^{2}+P_{3}N_{0}^{3})} \end{array}$$ (1)

The polynomial function was fitted, and parameters were estimated using the maximum likelihood method (PROC CATMOD, SAS v9.4). The 6 data sets were fitted individually to Equation (1), and the type of functional response was determined by the signs of *P*_1_ and *P*_2_. If the linear coefficient is negative (*P*_1_ < 0), it describes a Type II functional response because the proportion of prey consumed declines monotonically with the initial prey density (Juliano 2001). If *P*_1_ > 0 and *P*_2_ < 0, the proportion of prey eaten is initially positively density-dependent and describes a Type III functional response.

Once the functional response type was determined, the second step involved estimating the functional response parameters (*T*_*h*_, and either *a* [for Type II] or *b*, *c*, and *d* [for Type III]) using nonlinear least squares regression (PROC NLIN, SAS v9.4). As experiments were conducted with prey depletion, an explicit deterministic model was used (Rogers 1972) (Equation 2), where *a* is the attack constant (or instantaneous search rate), *T* is the time that prey are exposed to the predator, and $T_{h}$ is the handling time


$$\begin{array}{l} N_{e}=\;N_{0}[1-exp\;(-a(T\;-\;T_{h}N_{e}))] \end{array}$$ (2)

associated with each prey consumed. Equation (2) utilizes a constant attack rate (*a*) and describes a Type II functional response. A Type III functional response occurs when the attack rate is a function of prey density and can be modeled using a general useful form where *a* is a hyperbolic function of *N* (Juliano 2001). The simplest form of the Type III functional response model incorporates prey depletion (Hassel et al. 1977) and can be written as:


$$\begin{array}{l} N_{e}=N_{0}\left\{1-exp\left[(d+bN_{0})(T_{h}N_{e}-T)/(1+cN_{0})\right]\right\} \end{array}$$ (3)

where *b*, *c*, and *d* are constants that relate *a* and *N*_0_: $a=(d+bN_{0})/(1+cN_{0})$.

Functional response model parameters were removed if not significantly different from 0 (i.e., 95% confidence intervals included 0), and the model was recalculated until the minimal model was achieved.

To compare Type II functional responses between the different predator/prey stage groups, an equation with indicator variables was utilized (Juliano 2001):


$$\begin{array}{l} N_{e}=N_{0}\left\{1-exp\left[(\hat{a}+{\hat{D}}_{a}[\;j])\;[\;({\hat{T}}_{h}+{\hat{D}}_{Th}(\;j)N_{e}-T)]\right]\right\} \end{array}$$ (4)

where *j* is an indicator variable that takes value 0 for group 1 and 1 for group 2. The parameters ${\hat{D}}_{a}$ and ${\hat{D}}_{Th}$ estimate the differences in individual parameter values *a* and *T*_*h*_, respectively, between groups. If these parameters are significantly different from 0, then the 2 groups will differ significantly in the corresponding parameters. Nonlinear least squares regressions were again used to obtain parameter estimates. Comparisons of *a* and *T*_*h*_ were only conducted between the same predator stage feeding on different whitefly stages and between different predator stages feeding on the same whitefly stage. Predator performance in terms of *a* and *T*_*h*_ was compared by conducting Student’s *t*-tests for the null hypothesis that ${\hat{D}}_{a}$ and ${\hat{D}}_{Th}$ = 0 followed by Bonferroni’s post hoc tests (*α* = 0.0055).

## Results

**Fig. 1.**
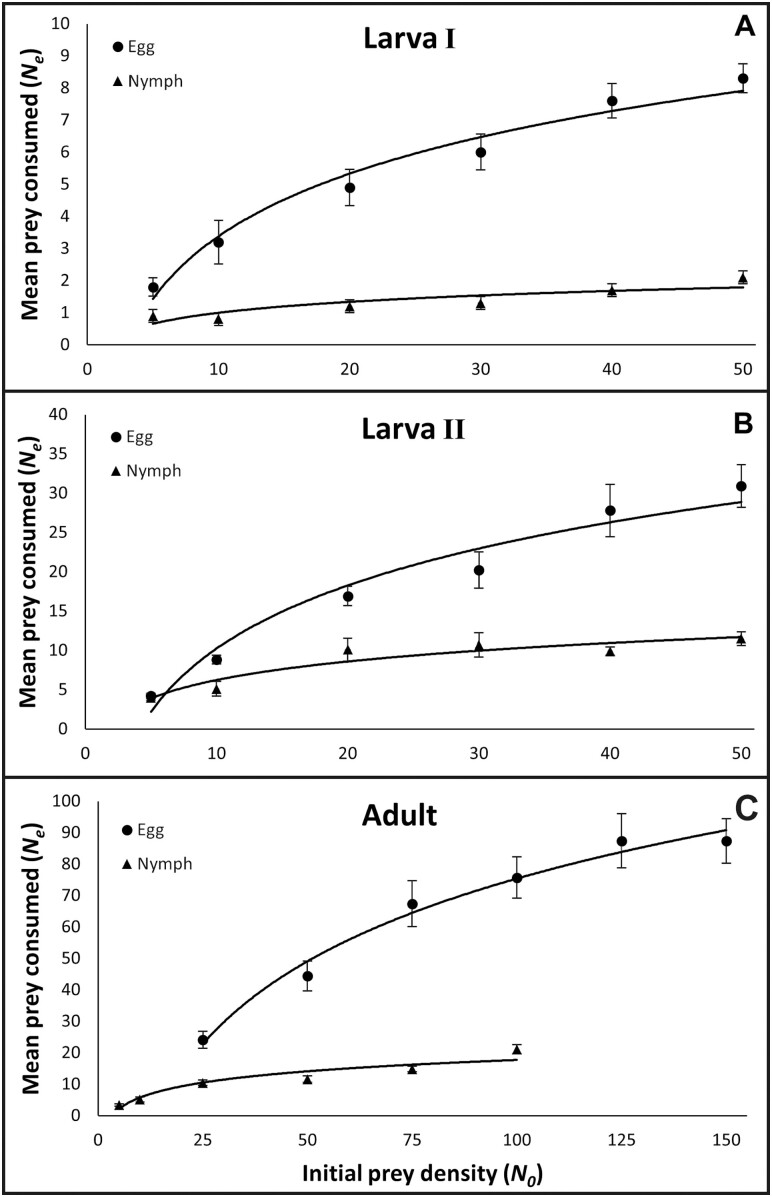
Functional responses of *Franklinothrips vespiformis* A) first- and B) second-instar larvae or adults C) when exposed to varying densities of *Bemisia tabaci* (MEAM1) eggs or nymphs. Points represent means ± SE of observed values (*N* = 10, *t* = 24 h).

Results of the logistic regression analyses showed a significantly negative linear coefficient (*P*_1_ <** **0) and positive quadratic coefficient (*P*_2_ > 0) for all *F. vespiformis* larval stage treatments (Table 1), indicating Type II functional responses with declining proportion of prey eaten as prey densities increased (Fig. 1A and B). This was also the case for *F. vespiformis* adults feeding on *B. tabaci* eggs and nymphs (Fig. 1C). The cubic coefficient (*P*_3_) was not a significant term in any of the logistic regression models. The maximum daily prey consumption or attack rate (*T*/*T*_*h*_) for eggs was calculated as 13.63, 55.41, and 108.10 for first-stage larvae, second-stage larvae, and adults, respectively. The maximum attack rate for nymphs was calculated as 2.55, 13.16, and 28.18 for first-stage larvae, second-stage larvae, and adults, respectively (Table 2). The handling times (*T*_*h*_) of *F. vespiformis* larvae and adults ranged from 0.22 to 1.76 h for *B. tabaci* eggs and 0.85–9.39 h for *B. tabaci* nymphs (Table 2). The random-predator equation fitted the observed data well for all predator–prey stage combinations (see *R*^2^ coefficients, Table 2).

**Table 1.** Maximum likelihood estimates from logistic regressions of the proportion of *Bemisia tabaci* (MEAM1) life stages consumed by various *Franklinothrips vespiformis* stages as a function of initial densities

		*F. vespiformis* larvae I			*F. vespiformis* larvae II			*F. vespiformis* adult		
Prey stage	Parameters	Estimate ± SE	χ^2^	*P*-value	Estimate ± SE	χ^2^	*P*-value	Estimate ± SE	χ^2^	*P*-value
Egg	Intercept (*P*_o_)	−0.3779 ± 0.2905	1.69	0.1934	2.4227 ± 0.3798	40.7	<0.0001	3.6554 ± 0.3379	117.06	<0.0001
	Linear (*P*_1_)	−0.0451 ± 0.0211	4.57	0.0326	−0.0618 ± 0.0241	6.56	0.0105	−0.0267 ± 0.0066	16.62	<0.0001
	Quadratic (*P*_2_)	0.0004 ± 0.0003	1.47	0.2250	0.0005 ± 0.0004	1.71	0.1908	0.0000 ± 0.0000	1.04	0.3082
Nymph	Intercept (*P*_o_)	−1.3431 ± 0.4414	9.26	0.0023	1.2423 ± 0.2848	19.03	<0.0001	0.7561 ± 0.1831	17.03	<0.0001
	Linear (*P*_1_)	−0.0936 ± 0.0353	7.04	0.0080	−0.0818 ± 0.0199	16.84	<0.0001	−0.0552 ± 0.0070	63.06	<0.0001
	Quadratic (*P*_2_)	0.0012 ± 0.0006	3.96	0.0466	0.0011 ± 0.0003	12.16	0.0419	0.0003 ± 0.0001	37.02	<0.0001

**Table 2.** Estimated mean ± SE of parameters attack rate (*a*) and handling time (*T*_*h*_) of *Franklinothrips vespiformis* on different life stages of *Bemisia tabaci* (MEAM1)

Stage					Asymptotic 95% CI				Asymptotic 95% CI			
Predator	Prey	Type	*a* (h^−1^)	SE	Lower	Upper	*T*_*h*_ (h)	SE	Lower	Upper	*T*/*T*_*h*_	*R* ^2^
Larva I	Egg	II	0.0188	0.0036	0.0115	0.0261	1.7606	0.3248	1.1104	2.4109	13.63	0.929
	Nymph	II	0.0057	0.0023	0.0012	0.0103	9.3989	2.3445	4.7059	14.092	2.55	0.753
Larva II	Egg	II	0.0911	0.0320	0.0271	0.1551	0.4331	0.1559	0.1211	0.7452	55.41	0.914
	Nymph	II	0.0779	0.0293	0.0193	0.1366	1.8243	0.2110	1.4020	2.2466	13.16	0.886
Adult	Egg	II	0.2162	0.1069	0.0023	0.4301	0.2221	0.0341	0.1537	0.2904	108.1	0.936
	Nymph	II	0.0261	0.0050	0.0161	0.0362	0.8517	0.1221	0.6072	1.0962	28.18	0.933

Values of *T*/*T*_*h*_ represent the maximum daily prey consumption rate, and *R*^2^ is the coefficient of determination.

Handling time and attack rate coefficient estimates are presented in Table 2, and comparisons of these estimates between predator–prey combinations using the indicator variable equation are presented in Table 3. When comparing the same predator stage feeding on *B. tabaci* eggs or nymphs, first-stage larvae exhibited higher attack rates on eggs (*t* = 2.835, df = 116, *P* = 0.0054). No differences in attack rates between prey stages were observed for second-stage larvae (*t* = 0.254, df = 116, *P* = 0.8000) or adults (*t* = 2.397, df = 116, *P* = 0.0189). Comparing different predator stages feeding on the same *B. tabaci* stage, there was a significant difference in attack rates exhibited between first- and second-stage larvae feeding on eggs (*t* = 2.824, df = 116, *P* = 0.0055) and first- and second-stage larvae feeding on nymphs (*t* = 3.223, df = 116, *P* = 0.0016). There were no observed differences in attack rates for first-stage larvae and adults feeding on eggs (*t* = 2.431, df = 116, *P* = 0.0166), first-stage larvae and adults feeding on nymphs (*t* = 2.609, df = 116, *P* = 0.0103), second-stage larvae and adults feeding on eggs (*t* = 1.189, df = 116, *P* = 0.2368), or second-stage larvae and adults feeding on nymphs (*t* = 1.721, df = 116, *P* = 0.0879) (Table 3).

**Table 3.** *D*_*a*_ and *D*_*Th*_ values were estimated using the indicator variable equation comparing attack and handling times within and between *Franklinothrips vespiformis* life stages preying on different *Bemisia tabaci* (MEAM1) stages

Stage				Asymptotic 95% CI					Asymptotic 95% CI	
Comparison	Parameter	Estimate	SE	Lower	Upper	Parameter	Estimate	SE	Lower	Upper
FL1-BE | FL1-BN	*D*_*a*_	−0.0131^a^	0.0046	−0.0222	−0.0039	*D*_Th_	7.6381	3.7249	0.2605	15.0156
FL1-BE | FL2-BE	*D*_*a*_	0.0723^a^	0.0256	0.0217	0.1229	*D*_Th_	−1.3275	0.9387	−3.1867	0.5316
FL1-BE | FA-BE	*D*_*a*_	0.1974	0.0812	0.0366	0.3582	*D*_Th_	−1.5385	2.5830	−6.6545	3.5775
FL1-BN | FL2-BN	*D*_*a*_	0.0722^a^	0.0224	0.0279	0.1165	*D*_Th_	−7.5737	6.9073	−21.2546	6.1071
FL1-BN | FA-BN	*D*_*a*_	0.0204	0.0078	0.0049	0.0359	*D*_Th_	−8.5469	7.0992	−22.6078	5.5140
FL2-BE | FA-BE	*D*_*a*_	0.1251	0.1052	−0.0833	0.3335	*D*_Th_	−0.2110	0.3338	−0.8722	0.4501
FL2-BE | FL2-BN	*D*_*a*_	−0.0132	0.0520	−0.1162	0.0899	*D*_Th_	1.3912^a^	0.3496	0.6987	2.0837
FL2-BN | FA-BN	*D*_*a*_	−0.0518	0.0301	−0.1115	0.0079	*D*_Th_	−0.9726^a^	0.2457	−1.4592	−0.4861
FA-BE | FA-BN	*D*_*a*_	−0.1901	0.0793	−0.3471	−0.0304	*D*_Th_	0.6296	0.4747	−0.3106	1.5698

Combinations are abbreviated: Predator stage *Franklinothrips* “F” (L1 = larvae 1, L2 = larvae 2, A = adult)—prey stage *Bemisia* “B” (E = egg, N = nymph). Vertical bars separate the predator:prey stage combinations being compared.

^a^Statistical significance that *D*_*a*_ or *D*_*Th*_ ≠ 0 following Bonferroni’s correction (*α* = 0.0055).

Comparing the handling times of the same predator stage feeding on *B. tabaci* eggs or nymphs showed that second-stage larvae exhibited longer handling times feeding on nymphs (*t* = 3.979, df = 116, *P* = 0.0001), but no differences in handling times between prey stages was observed for first-stage larvae (*t* = 2.051, df = 116, *P* = 0.0425) or adults (*t* = 1.326, df = 116, *P* = 0.1874) (Table 3). Comparing the handling times of different predator stages feeding on the same *B. tabaci* stage showed second-stage larvae feeding on nymphs exhibited higher handling times than adults (*t* = 3.958, df = 116, *P* < 0.0001) (Table 3). There were no observed differences in handling times for first- and second-stage larvae feeding on eggs (*t* = 1.414, df = 116, *P* = 0.0800), first-stage larvae and adults feeding on eggs (*t* = 0.596, df = 116, *P* = 0.2763), first- and second-stage larvae feeding on nymphs (*t* = 1.096, df = 116, *P* = 0.1377), first-stage larvae and adults feeding on nymphs (*t* = 1.204, df = 116, *P* = 0.1155), and second-stage larvae and adults feeding on eggs (*t* = 0.632, df = 116, *P* = 0.2643) (Table 3).

## Discussion

The results from this study for the first time demonstrated that both larvae and adults of *F. vespiformis* were voracious predators of the immature stages of *B. tabaci* and highlighted its potential efficacy as a biological control agent for this pest. For each of the predator–prey stage relationships examined, a Type II functional response provided a good fit to the data. Our results are similar to findings for many generalist invertebrates, where similar foraging effort is exerted at both low and high prey densities (Kalinkat et al. 2023). However, the type of functional response can change due to differences in response parameters under various predator–prey dynamics and environmental conditions. Some of these factors include prey body mass and defense capabilities (Hammill et al. 2010, Vucic-Pestic et al. 2010), environmental temperature (Englund et al. 2011, DeLong et al. 2023), and habitat complexity (Kreuzinger-Janik et al. 2019). Depending on the desired outcome of specific biological control programs, a Type II functional response may be more useful than a Type III response and vice versa. Type II functional responses can lead to destabilized predator–prey dynamics when there is high prey exploitation at low prey densities, while Type III can stabilize systems by providing prey refuge at low densities (Williams and Martinez 2004). Thus, if a rapid curative result of prey infestations is desired just prior to taking a crop to market, then the release of a biological control agent with a Type II functional response may be preferred, but predators could eventually starve, and frequent reintroductions will be needed to maintain low pest densities. Further studies utilizing *F. vespiformis* as a biological control agent in both a curative and preventative manner need to be performed under various production system conditions to fully inform growers of the best practices for its use.

Toward the development of *F. vespiformis* in a biocontrol program for *B. tabaci*, it exhibits several positive attributes but potentially some challenging ones as well, which we will discuss in relation to other natural enemies. Compared to parasitoids, the utility of generalist predators such as *B. tabaci* biological control agents is less understood (Kheirodin et al. 2020). The role of parasitoids in managing *B. tabaci* populations has been documented for multiple species, and their use has seen wide adoption by growers over the last few decades (Gerling et al. 2001, Naranjo 2001, Lahey and Stansly 2015, Liu et al. 2015). While parasitoids can offer viable biological control options for *B. tabaci*, they are limited to parasitizing the nymphal stages, which may result in delayed *B. tabaci* population decline and additional crop loss. The inability to complete development without hosts can make parasitoid populations susceptible to high mortality levels during periods of low host densities (Bompard et al. 2013), especially in greenhouses where their ability to migrate and seek alternative hosts is limited. Unlike parasitoids, generalist predators such as *F. vespiformis* typically feed on multiple *B. tabaci* life stages and may be able to utilize nonprey resources during periods of low target prey densities, and these traits in some systems could offer a more rapid and persistent biological control solution. Despite their ability to utilize alternative resources, biological control programs utilizing large generalist predators appear to pose some challenges in controlled environments due to their high developmental prey requirements and the amount of supplemental food needed to ensure population persistence when prey densities are low. In greenhouse settings, our previous study observed that populations of *F. vespiformis* are prone to decline during periods of low prey densities unless supplemental food is provided in large amounts (Schoeller et al. 2022). Thus, curative rather than preventive releases may be a more effective tactic for this species. This is important because influxes of large populations of whiteflies often occur when crops are harvested in the vicinity of greenhouses, which is a common problem in large parts of the southeastern United States. Previous studies highlighting the efficacy of generalist predators compared to parasitoids as biological control agents (e.g., Symondson et al. 2002, Stiling and Cornelissen 2005) demonstrate the need for additional research to assess whether generalist predators offer management options that are comparable to or work synergistically with parasitoids for controlling *B. tabaci*.

One of the generalist predator taxa that have seen the most utility as biological control agents for *B. tabaci* are phytoseiid mites (Nomikou et al. 2001), and multiple studies have examined their functional responses to *B. tabaci* life stages. For example, Han et al. (2020) observed that adult female *Neoseiulus bicaudus* (Wainstein) had maximum daily consumption rates of approximately 11 and 6 individuals for eggs and second-instar nymphs, respectively. This was higher than the max daily consumption rate of 9 eggs and 3 second-instar nymphs observed for *Neoseiulus cucumeris* (Oudemans) (Li et al. 2017) but lower than *Amblyseius tamatavensis* Blommers, which consumed an average of 21 *B. tabaci* eggs per day on various host plants (Barbosa et al. 2019). These studies are just a few examples that suggest *F. vespiformis* has higher maximum daily predation than phytoseiid mites in *B. tabaci* life stages. One factor that makes it difficult to predict whether the high daily predation maximum of *F. vespiformis* predisposes it to being a more efficient biological control agent compared to mites is the fact predatory mites typically have very high population numbers, which could offset having lower daily maximum predation rates.

Another important group of *B. tabaci* generalist predators that have seen utility as biological control agents are coccinellid beetles. The genus *Delphastus* contains 3 species that are key predators of *B. tabaci*: *D. catalinae* (Horn), *D. pallidus* LeConte, and *D. pussilus* LeConte (Hoelmer et al. 1994, Heinz et al. 1999, Razze et al. 2016, Kumar et al. 2020). Most studies have focused on the use of *D. catalinae* (frequently misidentified as *D. pussilus* in earlier studies) due to its commercial availability (Hoelmer and Pickett 2003). Kumar et al. (2020) compared the functional responses of *D. catalinae* and *D. pallidus* on *B. tabaci* eggs and found that *D. catalinae* had higher maximum attack rates on biotypes Q and B (65–79 eggs/day) than *D. pallidus* (55 eggs/day). These values are comparable or slightly higher to those observed for *F. vespiformis* second-stage larvae feeding on biotype B eggs but lower than those observed for adults. Given their small size (ca. 1.5 mm), *Delphasus* beetles appear to prefer whitefly eggs over nymphs (Kumar et al. 2020), and this preference for eggs could favor the use of the much larger *F. vespiformis* for biological control, which can inflict greater mortality on the nymphal stages, but their combined use is also an option. Some species of coccinellids are notorious for migrating away from the point of release (Obrycki and Kring 1998), which is not as big of an issue in greenhouses but could impact biological control efforts in the field. Our previous work on lima beans suggests that *F. vespiformis* adults are relatively stationary and prefer not to fly (Schoeller et al. 2022), which could be another beneficial trait as a biological control agent but would need to be investigated in more systems.

Commercial availability of biological control agents for growers needs to be an important consideration for any species being tested and is crucial for their successful implementation (van Lenteren 2012). Determining optimal *F. vespiformis* release strategies is necessary to focus commercial production on a product that has the best chance of limiting crop damage by the target pest. The available information on *F. vespiformis* biology suggests that commercial production of adults may be the most economical strategy. The total activity period of the 2 larval stages is approximately 4 days compared to up to 60 days for adult female *F. vesipformis* (Hussain et al. 2022), making production targeting the use of larvae unattractive. We observed that adult *F. vesipiformis* possessed maximum attack rates at least 2-fold higher than first- and second-stage larvae and innundative releases of adults may provide the means for a rapid knock-down of *B. tabaci* populations. As mentioned previously, *F. vespiformis* produces a pupal cocoon, and adults remain inside this cocoon for 2–4 days postpupation. Hoddle et al. (2001) investigated the possibility of commercial harvesting of the cocoons of *Franklinothrips orizabensis* Johanson using parafilm cones with some success. We have had similar success harvesting *F*. *vespiformis* cocoons using rice husks and believe this may be a commercially viable method for the harvest and transport of adults. Additionally, Larentzaki et al. (2007a) examined the effects of cold storage on *F. vespiformis* survival, reproduction and development and concluded that cold storage of adults at 10–12 °C for 3–5 wk yielded satisfactory survival results, opening further possibilities for facilitating commercial production and subsequent transport of *F. vespiformis*.

The data from this study provide the foundation for examining this predator’s future as a whitefly biological control agent, and subsequent studies must address many important questions about its utility in greenhouse and field settings. Determining whether *F. vespiformis* will attack and reduce *B. tabaci* and other whitefly species on ornamental and vegetable crops under greenhouse conditions is the logical next step toward developing a biological control program, followed by studies refining the release rates of *F. vespiformis* for both preventative and curative pest management applications and how these rates may differ by target pest species, crop, and environmental conditions. Very little information also exists on *F. vespiformis* prey preferences and how it may respond to the presence of alternative pest species and subsequently, the level of whitefly control that can be achieved in multiprey systems.
